# Impact of the variations in potential glycosylation sites of the hemagglutinin of H9N2 influenza virus

**DOI:** 10.1007/s11262-018-1623-7

**Published:** 2018-12-29

**Authors:** Qianqian Peng, Rui Zhu, Xiaobo Wang, Huoying Shi, Matthew Bellefleur, Shifeng Wang, Xiufan Liu

**Affiliations:** 1 College of Veterinary Medicine, Yangzhou University, Yangzhou, 225009 Jiangsu China; 2 Key Laboratory of Avian Preventive Medicine, Ministry of Education, Yangzhou University, Yangzhou, China; 3 Jiangsu Key Laboratory of Preventive Veterinary Medicine, Yangzhou University, Yangzhou, China; 4 Key Laboratory of Animal Infectious Diseases, Ministry of Agriculture, Yangzhou University, Yangzhou, China; 5 Department of Infectious Diseases and Immunology, College of Veterinary Medicine, University of Florida, Gainesville, FL 32611-0880 USA; 6 Jiangsu Co-innovation Center for the Prevention and Control of Important Animal Infectious Diseases and Zoonoses, Yangzhou, 225009 China

**Keywords:** H9N2, Glycosylation sites, Hemagglutinin, Aa residue 218, Aa residue 313

## Abstract

Variations in the potential glycosylation sites were observed in hemagglutinin (HA) sequences of H9N2 avian influenza virus isolated in China, deposited in the Influenza Virus Resource of NCBI before 2017, which showed a deleted glycosylation site at amino acid residue 218, and an introduced glycosylation site at amino acid residue 313. Based on the variations in the glycosylation sites at these amino acids, H9N2 avian influenza viruses could be divided into three categories. Firstly, most of the H9N2 influenza viruses were 218G^+^ viruses; less 313G^+^ viruses were isolated between 1997 and 2004. Secondly, the occurrence of the 218G^+^/313G^+^ viruses increased, while the 218G^+^/313G^−^ viruses decreased from 2005 to 2012. Thirdly, from 2013 to 2016, the 218G^−^/313G^+^ viruses were predominant compared to the 218G^+^/313G^+^ viruses. Here, based on an F/98 virus backbone, a 218G^+^/313G^−^ virus, two reassortment viruses were generated, and named rF/HA218G^+^/313G^+^ and rF/HA 218G^+^/313G^−^, respectively. HA protein migration demonstrated that the glycosylation sites at amino acid residues 313 and 218 were both functional. The absence of the glycosylation site at amino acid residue 218 and the presence of the glycosylation site at amino acid residue 313 increased antibody binding and moderately prevented the virus from escaping neutralization with homologous antisera. Additionally, compared to the F/98 virus (218G^+^/313G^−^), the viruses rF/HA218G^+^/313G^+^ or rF/HA218G^−^/313G^+^ showed significantly increased infectivity of MDCK cells, chicken embryo eggs, and trachea and lung tissue of SPF chickens, but did not display differences in airborne spread in chickens or infectivity of mice compared with its parental virus F/98.

## Introduction

The H9N2 avian influenza virus was first detected in the North American turkey in 1966, after which it spread throughout the world, causing huge economic losses in the poultry industry. The antigenic structure of the H9N2 influenza virus has been changing all the time, including changes in N-linked glycosylation (NLG) sites of the hemagglutinin (HA) protein [1]. NLG is a specific post-translational modification of viral surface glycoproteins, HA and neuraminidase (NA), whereby oligosaccharides are attached through N-glycosidic linkages to the Asn residue of the glycosylation motif Asn-X-Ser/Thr-X, where X may represent any amino acid except proline [2]. NLGs from HA protein play an important role on a further structural and functionality modification of influenza A virus (IAV). Glycosylation is essential for protein folding and maturation through the endoplasmic reticulum (ER) and golgi apparatus [3]. Changes in the number or location of NLG sites in the spherical head of HA protein can affect the biological activity of IAV [4]. NLGs of HA protein regulate the virulence of IAV by adjusting the biological activity of HA in the IAV [5–8]. Furthermore, NLGs allow IAV to evade host antibody recognition [9, 10]. The NLG status of the receptor-binding domain of HA in IAV mediates protective antibody responses against the 1918 and 2009 pandemic H1N1 viruses [11, 12]. Modifying glycosylation sites, especially in the stalk domain, have been explored to broaden the breadth of antibody responses induced by vaccination [13]. HA protein of the IAV is the primary target for neutralizing antibody recognition. NLGs have been shown to shield the antigenic sites in HA and promote the evolution of the virus [1]. Moreover, addition of a NLG was associated with resistance to neutralizing or enhancing growth in vaccinated mice [14]. And, the receptor binding avidity through the addition of NLGs to the HA globular domain was modulated to maintain fitness during antigenic evolution [15].

HA protein of IAV is the major target recognized by neutralizing antibodies and glycans have been proposed to shield antigenic sites on HA, thereby promoting virus survival in the face of widespread vaccination and/or infection [1, 8, 16]. The variations in the glycosylation (abbreviates G) sites at amino acid residues 218 (named 218G^+^, Asn-Arg-Thr-Phe, NRTF) and 313 (named 313G^+^, Asn-Cys-Ser-Lys, NCSK) emerged in the process of the evolution of H9N2 avian influenza viruses. There are four phenotype including 218G^+^/313G^−^, 218G^+^/313G^+^, 218G^−^/313G^+^, and 218G^−^/313G^−^. 218G^+^ means the glycosylation site at amino acid residues 218; in contrast, 218G^−^ means no glycosylation site at amino acid residues 218, which are similar to 313G. And we propose the hypotheses that the variations in NLG sites of HA protein in H9N2 influenza virus might affect the biological characteristics of the H9N2 influenza virus. In this study, two reassortment viruses were generated by the reverse genetics system of influenza virus based on the F/98 virus backbone, a 218G^+^/313G^−^ virus, and named rF/HA218G^+^/313G^+^ and rF/HA 218G^+^/313G^−^, respectively. The effect of the variations in NLG sites on the H9N2 influenza virus was explored in antibody binding, and infectivity of embryonated eggs of specific-pathogen-free (SPF), MDCK cells, or SPF chickens.

## Materials and methods

### Ethics statement

All animal experiments were approved by the Jiangsu Administrative Committee for Laboratory Animals (permission number SYXK-SU-2007-0005) and complied with the Jiangsu Laboratory Animal Welfare and Ethics guidelines of the Jiangsu Administrative Committee of Laboratory Animals. All experiments were performed under biosafety level 2 (BSL2). All generated viruses included the variations in the glycosylation sites at amino acid residues 313 and 218, which had been found in nature, and not created artificially.

### Virus, cell, and sequence analysis of the HA gene of the H9N2 virus

The H9N2 virus, A/Chicken/Shanghai/F/98 (H9N2, Ck/SH/F/98; abbreviated as F/98), was isolated in Shanghai in 1998, stored at − 70 °C at the Animal Infectious Disease Laboratory, School of Veterinary Medicine, Yangzhou University, and identified by the National Influenza Center as the H9N2 subtype influenza virus [17, 18]. The GenBank accession numbers of the full gene sequence of the F/98 strain are AY253750-AY253756 and AF461532 [17]. Human embryonic kidney cells (293T) and Madin-Darby canine kidney (MDCK) cells, purchased from ATCC (Manassas, VA, USA), were maintained in Dulbecco’s modified Eagle’s medium (DMEM) (Sigma, St. Louis, MO, USA) supplemented with 10% fetal calf serum (Hyclone, South Logan, UT, USA) and were incubated at 37 °C with 5% CO_2_.

HA protein sequences of the H9N2 avian influenza viruses collected in China from 1998 to 2016, published before 2017 (date to Jan 1, 2017), were downloaded from the Influenza Virus Resource of NCBI (https://www.ncbi.nlm.nih.gov/genomes/FLU/Database/nph-select.cgi?go=database). After collapsing identical sequences, about 1900 HA sequences were available. The variations in potential NLG sites in HA protein were analyzed using DNAstar5.0 software (DNAstar, Madison, WI, USA).

### Cloning and generation of viral strains by reverse genetics

The primers, synthesized by Sangon Biotech (Shanghai) Co., Ltd. (Shanghai, China), used to amplify the DNA sequence to add the NLG site at amino acid residue 313 or to delete the NLG site at amino acid residue 218 in HA protein of F/98 virus were designed using Primer 5.0 software (Primer-E Ltd., Plymouth, UK), based on the HA gene sequence of the F/98 H9N2 avian influenza virus (primer sequences (5′–3′): HA-F, GTCGACCTCCGAAGTTGGGGGGGAGCAAAAGCAGGCGAATTTCA; HA-R, GGCATTTTGGGCCGCCGGGTTATTAGTAGAAACAAGGGTGTTTTT; 313G^+^-F, TTTGGAAACTGCTCAAAGTATGTTG; 313G^+^-R, CAACATACTTTGAGCAGTTTCCAAA; 218G^−^-F, ATATAAATAGAGTCTTCAAACCAAT; 218G^−^-R, ATTGGTTTGAAGACTCTATTTATAT). The full-length HA genes containing either the 313G^+^ mutation or the 218G^−^/313G^+^ mutations were amplified by PCR, and inserted into the transcriptional/expression vector pHW2000 gifts from Webster RG from St. Jude Children’s Research Hospital [19], resulting in the plasmids pHW204-HA218G^+^/313G^+^ and pHW204-HA218G^−^/313G^+^, respectively. Seven transcription/expression plasmids, including pHW201-PB2, pHW202-PB1, pHW203-PA, pHW205-NP, pHW206-NA, pHW207-M, and pHW208-NS, from the F/98 virus strain, were stocked in our lab at − 70 °C [18]. A total weight of 2.4 ng of the eight plasmid mixture with a certain weight of 1:1 was mixed with 100 µL Opti-MEM medium (GIBCO, BRL, Grand Island, USA). Next, 7 µL of PolyFect transfection reagent (QIAGEN, Duesseldorf, Germany) was added, and the samples were incubated at room temperature for 10 min and then added to the 80% confluent monolayers of 293T cell in 24-well plates. After incubation at 37 °C with 5% CO_2_ for 6 h, 2 µg/mL of tosylamide-phenyl-chloromethyl-ketone (TPCK)-trypsin (Sigma, St. Louis, MO, USA) was added to the wells. Thirty hours after transfection, the supernatants were harvested and inoculated into 10-day-old SPF embryonated chicken eggs for virus propagation. The rescued viruses were analyzed with a hemagglutinin assay, and the HA genes from the rescued viruses were sequenced by the Sangon Biotech (Shanghai) Co., Ltd. (Shanghai, China) to confirm the accuracy of the designed mutations.

### Hemagglutinin-inhibition (HI) assay

The HI assay was performed with an initial dilution of 1:10 according to standard methods [20]. The serum of the F/98 H9 avian influenza virus was collected from blood of three SPF chickens in our laboratory. Newcastle disease virus (NDV) and H5 subtype AIV sera were provided by the Animal Infectious Disease Laboratory of Yangzhou University, and 1% (vol/vol) solution of chicken red blood cells was used to carry out the assay [20]. Sera were treated for 18 h with RDE (a cholera leachate) (Sigma, St. Louis, MO, USA) to remove nonspecific inhibitors.

### Western blotting analyses

Viruses were propagated in the allantoic cavity of SPF chicken eggs and then purified by 20% sucrose gradient centrifugation at 30,000 rpm for 3 h. The concentrated virus was re-suspended in phosphate-buffered saline (PBS). Western blotting was performed to analyze the samples. Antisera against the F/98 virus in chicken was used as a primary antibody, and horseradish peroxidase-conjugated rabbit anti-chicken immunoglobulin G (IgG) (Abcam, Cambridge, MA) was used as a second antibody [21]. Binding was visualized with a chemiluminescent substrate using the kit ECL Plus Western Blotting System (GE Health-care, Chalfont St Giles, UK) according to manufacturer’s instructions. The quantitative analysis on movement was conducted using ImageJ program.

### Enzyme-linked immunosorbent assays (ELISAs)

Sucrose gradient-purified viruses were diluted in PBS and added to Nunc-Immuno MaxiSop 96-well plates (Corning, NY, USA) at 16 hemagglutinating units (HAU) per well. After incubation overnight at 4 °C, samples in wells were blocked with PBS-nonfat dry milk. Antisera against the F/98 virus in chicken were then added in serial twofold dilutions with PBS containing 0.05% Tween-20, and incubated for 3 h at 37 °C. After washing, goat anti-chicken horseradish peroxidase antibody (Abcam, Cambridge, MA) was added and allowed to incubate for 1.5 h at 37 °C. After washing, TMB (3,3′,5,5′-Tetramethylbenzidine) (Sigma, St. Louis, MO, USA) substrate was added, and the reaction was stopped by adding H_2_SO_4_. Absorbance was recorded at 450 nm using an automated ELISA plate reader (model EL311SX; Biotek, Winooski, VT) [22].

### Determination of the 50% egg infectious dose (EID_50_)

Virus samples diluted in PBS containing four antibiotics at concentrations of 10^−4^ to 10^−11^ were inoculated into 10-day-old SPF chicken eggs, respectively. The allantoic fluid of the allantoic cavity of the eggs from 24 to 120 h post-infection was harvested and analyzed for HA titers. The EID_50_ of the virus was calculated according to the Reed–Muench formula [23].

### Determination of the 50% tissue cell infectious dose (TCID_50_)

The virus was diluted in DMEM without serum to a concentration of 10^−1^ to 10^−11^ and then added to MDCK cells in 96-well plates, respectively. After incubation at 37 °C with 5% CO_2_ for 1 h, the supernatants were removed. The plates were washed twice with PBS, and then 100 µL of DMEM was added to every well. After incubation at 37 °C with 5% CO_2_ for 72 h, the HA titers of the cell supernatants were analyzed. The virus titers were calculated according to the Reed–Muench formula [23].

### Chicken experiments

To evaluate the effect of NLG sites at amino acid residues 313 and 218 on the pathogenicity of the viruses in chickens, 21-day-old White Leghorn SPF chickens, obtained from Laboratory Animal Center of Yangzhou University, were infected intranasally and intratracheally with a dose of 10^6^ EID_50_ of each virus. 3 and 5 days post-infection, the chickens were dissected following euthanasia with CO_2_, and the trachea and lung tissues were collected and ground into 20% (*w*/v) suspension in 1 mL PBS. Dilutions of 10^−1^ to 10^−10^ were inoculated into 10-day-old SPF chicken eggs. The eggs that died within 24 h were discarded, and the allantoic fluids in the allantoic cavity of SPF chicken eggs from 24 to 120 h were harvested and the HA titers were analyzed. The virus titers of the tissues were calculated according to the Reed–Muench formula [23].

### Mouse experiments

As previous reported, the glycosylation in the globular head of HA can affect on the pathogenicity and antigenicity of H5N1 AIVs [24]. Although the parent virus F/98 cannot infect mice [25, 26], we wonder whether the glycosylation site changes at amino acid residues 313 and 218 impact the viral phenotype in mice. To evaluate whether the strains with variations of potential NLG sites at amino acid residues 313 and 218 could replicate in mice, 6-week-old female BALB/c mice purchased from the Experimental Animal Center of Yangzhou University were infected intranasally with 10^6^ EID_50_ of each virus in 50 µL of PBS. On days 3 and 5 after infection, the mice were dissected following euthanasia, and the trachea and lung tissues of the mice were collected and into 20% (*w*/v) suspension in 1 mL PBS. Dilutions of 10^−1^ to 10^−10^ were inoculated into 10-day-old SPF chicken eggs. The allantoic fluids in the allantoic cavity of the eggs were harvested from 24 to 120 h, and the HA titers were analyzed.

### The spread and immunogenic characteristics of the virus

To evaluate whether NLG sites at amino acid residues 313 and 218 affect the transmission route of the virus, 27 21-day-old SPF chickens were divided into three groups, labeled as F/98, 218G^+^/313G^+^, and 218G^−^/313G^+^ group. Each group was further divided into 3 subgroups with each consisting of three chickens: the inoculated subgroup, the direct-contact subgroup, and the airborne contact subgroup. On the first day post-inoculation (dpi), naive chickens were housed in the same cage with inoculated chickens for the direct-contact subgroup, and naive chickens were placed in a cage directly adjacent to an inoculated subgroup for the airborne contact subgroup, with a distance of 50 cm between cages. Chickens in the inoculated subgroup of each virus were inoculated orally, intranasally, and intratracheally with 0.2 mL of a 10^6^ EID_50_ of the virus in PBS. Tracheal and cloacal swabs were obtained from these chickens at 3, 5, 7, and 9 dpi, and the virus of the tracheal and cloacal swabs was titrated in 10-day-old SPF chicken eggs for EID_50_ counts [23].

To identify whether the variations in potential glycosylation site at amino acid residues 313 and 218 resulted in antigenic variation of the H9N2 virus, the cross-protection between F/98 and rF/HA218G^+^/313G^+^ or rF/HA218G^−^/313G^+^ was studied. A total of 12 three-week-old SPF chickens were divided into four groups: the PBS group, the F/98 group, the rF/HA218G^+^/313G^+^ group, and the rF/HA218G^−^/313G^+^ group. Each group has three chickens. Chickens in the PBS group were immunized with PBS as a control, and the chickens in the F/98, rF/HA218G^+^/313G^+^, and rF/HA218G^−^/313G^+^ groups were immunized with the emulsion vaccine of the F/98 strain. At 3 weeks post-inoculation, chickens were bled from the wing vein for sera and challenged intranasally and intratracheally with 10^6^ EID_50_ of the indicated virus. Chickens were monitored daily for morbidity and mortality after challenge. At day 3 and 5 post-challenge, tracheal and cloacal swabs from challenged chickens were collected in 1 mL of PBS containing antibiotics and, following one freeze–thaw cycle, were centrifuged at 3000 rpm for 10 min. Of the resulting supernatant, 0.2 mL was taken, and inoculated in 10-day-old SPF chicken eggs. Viral shedding in the trachea and cloacal was evaluated via HA titers of the allantoic cavity of SPF chicken eggs at day 5 post-inoculated according to the standard of HA ≥ 2^3^ [27].

### Statistics analysis

Data were presented as the geometric means and standard deviations for all assays. The Mann–Whitney U test (GraphPad Software, Inc., San Diego, CA) was used to evaluate the differences in anti-F/98 serum binding to different viruses. A P value of 0.05 was considered statistically significant. Means and SEM from triplicate samples are shown. The data are representative of three independent experiments.

## Results

### Analysis potential glycosylation sites of the HA in H9N2 influenza virus and the generation of the mutant viruses

**Fig. 1:**
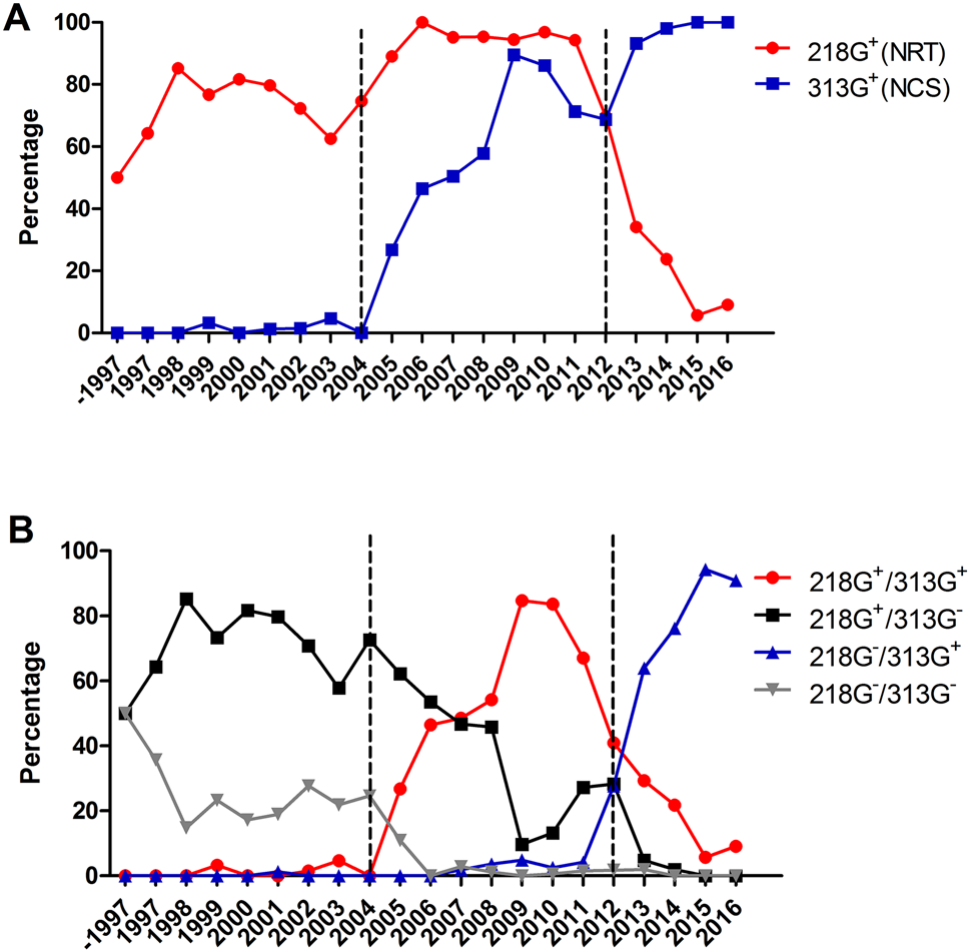
Analysis of 218 and 313 glycosylation sites in HA gene of H9N2 virus before 2017. About 1900 full-length H9N2 HA amino acid sequences deposited in GenBank from 1997 to 2016 (date to Jan 1, 2017) in China were analyzed. 218G^+^(NRT) indicates Asn-Arg-Thrat amino acid residues 218–220, a potential glycosylation site at amino acid residue 218; 313G^+^(NCS) indicates Asn-Cys-Serat amino acid residues 218–220, a potential glycosylation site at amino acid residue 218

To investigate the variations in NLG sites at amino acid residues 313 and 218, the HA sequences of the H9N2 avian influenza virus isolated in China, deposited in Influenza Virus Resource of NCBI (https://www.ncbi.nlm.nih.gov/genomes/FLU/Database/nph-select.cgi?go=database) before 2017 (date to Jan 1, 2017), were in statistics using DNAstar5.0 software (DNAstar, Madison, WI, USA) (Fig. 1). The proportion of the H9N2 313G^+^ viruses annually increased from 2004 to 2009 and have been predominant of more than 93% since 2013. The proportion of the 218G^+^ viruses were dominant over more than 89% from 2005 to 2011 and had decreased to lower than 35% from 2011 to 2016, and even lower than 10% during 2015–2016 (Fig. 1a). Of the variations in NLG sites at amino acids 218 and 313 of H9N2 influenza viruses, the 218G^+^/313G^−^ viruses were dominant over 50% before 2007. The 218G^+^/313G^+^ viruses were dominant over 40% from 2008 to 2012. The 218G^−^/313G^+^ viruses have been dominant over 63% from 2013 to 2016. The 218G^−^/313G^−^ viruses almost have disappeared since 2006 (Fig. 1b).

F/98 virus is a 218G^+^/313G^−^ virus. To understand the function of these variations in NGL sites between the amino acids 218 and 313 in the HA of the H9N2 influenza viruses last decade, two reassortant viruses, rF/HA218G^+^/313G^+^ and rF/HA218G^−^/313G^+^, were generated based on the F98 virus backbone. The HA titers of the allantoic fluids of eggs infected with rF/HA218G^+^/313G^+^ and rF/HA218G^−^/313G^+^ were 64 and 128, respectively (Table 1). The results of the HI assay showed that the two reassortant viruses, rF/HA218G^+^/313G^+^ and rF/HA218G^−^/313G^+^, viruses only reacted with F/98 serum, yet did not react with anti-NDV (Newcastle disease virus) and anti-H5 sera (Table 1). The results of the HA sequencing showed that the HA amino acid sequence of the rF/HA218G^+^/313G^+^ strain had NCSK at amino acid residues 313–316, indicating a increased NGL site in 313 site, and the HA amino acid sequence of the rF/HA218G^−^/313G^+^ strain had VRTF at amino acid residues 218–221 and NCSK at amino acid residues 313–316, indicating a decreased NGL site in 218 site, and a increased NGL site in 313 site, respectively.

**Table 1:** The HA titers and infectivity of viruses in SPF chicken eggs, cells, and the tissue of chickens

Virus	HA titers	HI titers			EID_50_/0.2 mL	TCID_50_/0.2 mL	Trachea^a^ (EID_50_/0.2 mL)		Lung^a^ (EID_50_/0.2 mL)	
		NDV	H5	F/98			3 days	5 days	3 days	5 days
F/98	2048	0	0	2560	10^−6.67^	10^−3.67^	10^−3.5^	10^−2.25^	10^−2.25^	10^−1.75^
rF/HA218G^+^/313G^+^	256	0	0	2560	10^−7.5*^	10^−4.52*^	10^−4.25*^	10^−2.75^	10^−2.75^	10^−2.25*^
rF/HA218G^−^/313G^+^	256	0	0	2560	10^−6.75#^	10^−3.75#^	10^−3.75#^	10^−2. 5^	10^−2.25^	10^−1.75#^

*Indicates that there is a significant difference between infectivity of rF/HA218G^+^/313G^+^ and F/98 strain in chicken embryo and cell

^#^Indicates that there is a significant difference between infectivity of rF/HA218G^+^/313G^+^ and rF/HA218G^−^/313G^+^ in chicken embryo and cell

^a^Virus mean titer in tracheae or lungs from three SPF chicken infected with each virus

### The effect of variations in the glycosylation sites at amino acid residues 313 and 218 on the mobility of HA protein

**Fig. 2:**
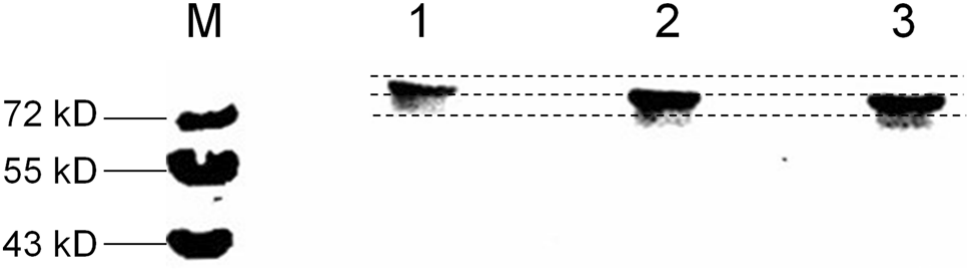
Western blot analysis of F/98, rF/HA218G^+^/313G^+^, and rF/HA218G^−^/313G^+^ viruses. The purified H9N2 viruses were incubated with chicken anti-F/98 antiserum. Binding was visualized with a chemiluminescent substrate using the kit ECL Plus Western Blotting System according to manufacturer’s instructions. Locations of marker proteins were indicated on the left, and the HA of AIV are indicated on the right. M: Protein Marker; 1: rF/HA218G^+^/313G^+^ virus; 2: rF/HA218G^−^/313G^+^ virus; 3: F/98 virus. The quantitative analyses of rF/HA218G^+^/313G^+^ virus, rF/HA218G^−^/313G^+^ virus, and F/98 virus on movement were 11307.447, 16895.861, and 17919.811, respectively, using ImageJ program

To determine whether amino acid residues 313 and 218 were functional NGL sites, the mobilities of HA proteins of the rF/HA218G^+^/313G^+^, rF/HA218G^−^/313G^+^, and F/98 strains were compared by western blotting. The results showed that the migration of HA protein of the rF/HA218G^+^/313G^+^ strain was significantly slower compared to the F/98 strain, whereas the migration of HA protein of the rF/HA218G^−^/313G^+^ strain was faster than that of the F/98 strain but slower than that of the rF/HA218G^+^/313G^+^ strain, indicating that the NGL sites at amino acid residues 218 and 313 of HA protein are functional glycosylation sites (Fig. 2) [21]. And the quantitative analysis of rF/HA218G^+^/313G^+^ virus, rF/HA218G^−^/313G^+^ virus, and F/98 virus on movement were 11307.447, 16895.861, and 17919.811, respectively, using ImageJ program.

### Variations in glycosylation sites at amino acid residues 313 and 218 physically in HA affect the antigenicity of H9N2 influenza virus

**Fig. 3:**
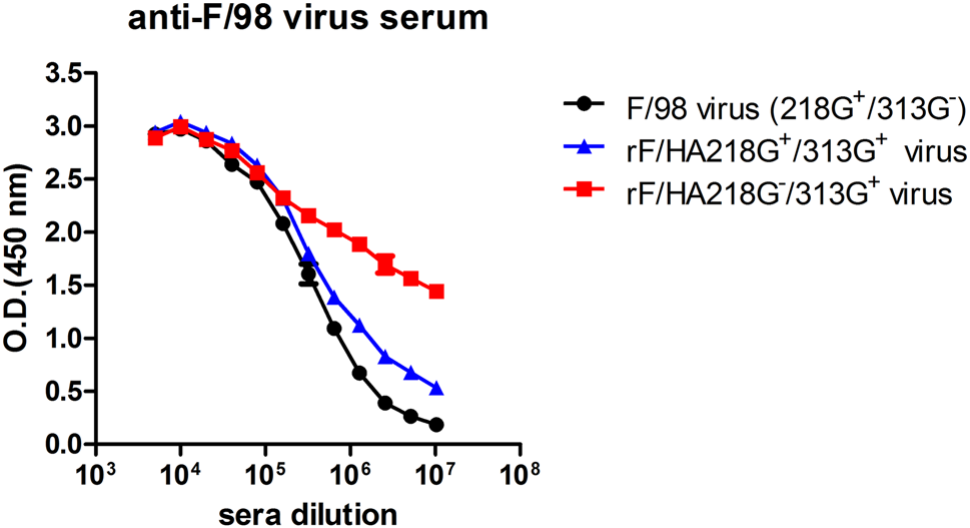
Variations in the glycosylation sites at amino acid residues 313 and 218 physically lead to antibody binding change. Antibody binding to different viruses was determined by ELISA using sera isolated from chickens infected with the F/98 virus possessing the glycosylation sites at amino acid residues 313. Means and SEM from triplicate samples are shown. The data are representative of three independent experiments. O.D., absorbance, recorded at 450 nm. *Indicated *p* < 0.05

To address if the variations in glycosylation sites at amino acid residues 313 and 218 in HA affect the antigenicity of H9N2 influenza virus, the antibody binding ELISAs were performed. Compared to the F/98 virus, the titer of the rF/HA218G^−^/313G^+^ virus binding to the sera of the F/98 virus was significantly stronger than the rF/HA218G^+^/313G^+^ virus, and the F/98 virus at the 10^−7^ diluent sera of the F/98 virus (Fig. 3), indicating that 313G^+^ with 218G^−^ could increase the binding ability of the rF/HA218G^−^/313G^+^ virus to the sera of the parental F/98, and affect the antigenicity of H9N2 influenza virus.

### The effect of variations in glycosylation sites at amino acid residues 313 and 218 on the biological characteristics of the H9N2 virus

To study the effect of HA glycosylation sites on the biological characteristics of the H9N2 virus, such as the 50% egg infectious dose (EID_50_), 50% egg lethal dose (ELD_50_), and 50% tissue cell infectious dose (TCID_50_), pathogenicity in chickens and mice, transmission route, and antigenic variation of the H9N2 virus in chickens were analyzed. Compared to the F/98 strain, the EID_50_ and TCID_50_ of the rF/HA218G^+^/313G^+^ strain was significantly increased by 6.76-fold, and 7.07-fold, respectively. Conversely, the EID_50_ and TCID_50_ of the rF/HA218G^−^/313G^+^ strain had similar titers but were significantly decreased by 5.62-fold, and 6.02-fold, respectively, relative to the EID_50_ and TCID_50_ of the rF/HA218G^+^/313G^+^ strain (Table 1). At days 3 and 5 post-infection, the EID_50_ titers of the rF/HA218G^+^/313G^+^ strain in chicken tissue were significantly higher by 5.62-fold and 3.16-fold in the trachea and decreased by 3.16-fold and 3.16-fold in the lung compared to the F/98 strain. However, the infectious doses of the rF/HA218G^−^/313G^+^ strain were decreased by 3.16-fold and 1.78-fold in the trachea and decreased by 3.16-fold and 3.16-fold in the lung compared to the rF/HA218G^+^/313G^+^ strain; however, there were no significant differences of EID_50_ and TCID_50_ in either the trachea or lung, compared to the F/98 strain (Table 1).

In mice, no clinical characteristics were observed, and none of the viral strains were detected in the lung at days 3 and 5 post-infection, which is also characteristic of the F/98 strain in mice. However, the discomfort, the mild diarrhea, and the mucus in the sinuses of the chickens infected with the viral strains were observed at days 3 post-infection, and then disappeared in the next several days.

Animal transmission experiments showed that the F/98, rF/HA218G^+^/313G^+^, and rF/HA218G^−^/313G^+^ strains could be spread by direct contact and airborne contact, indicating that the variations in the glycosylation sites at amino acid residues 313 and 218 had no effect on the transmission routes of the virus (Table 2).

**Table 2:** Results of viruses isolated from tracheal and cloacal swabs from 3 to 9 days. Three chickens were included in each way of infection with each virus

Virus	Ways of infection	Positive/total^a^							
		Tracheal swabs				Cloacal swabs			
		3 days	5 days	7 days	9 days	3 days	5 days	7 days	9 days
F/98 (HA218G^+^/313G^−^)	Directly inoculation	3/3	2/3	0/3	0/3	2/3	2/3	0/3	0/3
	Directly contact	2/3	2/3	0/3	0/3	0/3	1/3	0/3	0/3
	Airborne contact	0/3	2/3	1/3	0/3	0/3	0/3	0/3	0/3
rF/HA218G^+^/313G^+^	Directly inoculation	3/3	3/3	2/3	0/3	2/3	2/3	0/3	0/3
	Directly contact	2/3	2/3	1/3	0/3	0/3	1/3	0/3	0/3
	Airborne contact	0/3	2/3	1/3	0/3	0/3	0/3	0/3	0/3
rF/HA218G^−^/313G^+^	Directly inoculation	3/3	2/3	1/3	0/3	2/3	2/3	1/3	0/3
	Directly contact	1/3	2/3	1/3	0/3	0/3	1/3	1/3	0/3
	Airborne contact	0/3	1/3	1/3	0/3	0/3	0/3	0/3	0/3

^a^Number of positive tracheal or cloacal/total number of chickens. Positive sample indicated higher than the detection limit of 2^2^ HA titer

In the immunogenic test, it was found that the antibody (HI:128–512) induced by the F/98 vaccine could provide 100% protection against the challenge by both the rF/HA218G^+^/313G^+^ and rF/HA218G^−^/313G^+^ strains since no shedding was detected in the groups challenged with those strains. This result indicated that the variations in glycosylation sites at amino acid residues 313 and 218 did not result in the antigenic variation of the F/98 strain. In the control group, all chickens immunized with PBS exhibited viral shedding in the trachea or cloaca with 0 HI titer (Table 3).

**Table 3:** Isolation of H9N2 viruses in vaccinated chickens after challenged with PBS, F/98, rF/HA218G^+^/313G^+^, and rF/HA218G^−^/313G^+^ viruses at 3 and 5 days

Groups	Positive/total^a^			
	Tracheal swabs		Cloacal swabs	
	3 dpi	5 dpi	3 dpi	5 dpi
SPF chickens inoculated F/98 strain virus after immunized with PBS	3/3	2/3	2/3	2/3
SPF chickens inoculated F/98 strain virus after immunized with F/98 vaccine	0/3	0/3	0/3	0/3
SPF chickens inoculated rF/HA218G^+^/313G^+^ virus after immunized with F/98 vaccine	0/3	0/3	0/3	0/3
SPF chickens inoculated rF/HA218G^−^/313G^+^ virus after immunized with F/98 vaccine	0/3	0/3	0/3	0/3

Three chickens were included in each experiment group

^a^Number of positive tracheal or cloacal/total number of chickens. Positive sample indicated higher than the detection limit of 2^2^ HA titer

## Discussions

Normally N-linked glycosylation involves a class of amino acid constructs with N-X-S/T-X [28]. Changes in certain glycosylation sites of the viral proteins may have a significant effect on the biological characteristics of the virus [29–31], such as virulence, and the function of the affected viral proteins. Studies have shown that changes in the glycosylation status near HA protein cleavage site may affect the virulence of the virus [7]. Changes of the glycosylation status in the antigenic region of HA protein of the influenza virus may affect the binding of the virus to the antibody to generate the mutants in HA [6, 32], while changes of the glycosylation at some positions have no effect on the structure and function of the virus.

Based on variations in glycosylation sites at amino acid residues 313 and 218, the evolution of H9N2 avian influenza viruses are divided into three categories: 1997–2004, 2005–2012, and 2013–2016. During these categories, variations in the glycosylation sites at amino acid residues 313 and 218 were dynamic. In last decade, the proportion of the 218G^+^/313G^+^ viruses and the 218G^−^/313G^+^ viruses were predominant during 2008–2012, 2013–2016, respectively. These changes occur through the process of viral evolution. Based on the viruses from the latest two categories (from 2008 to 2012, and from 2013 to 2016), two F/98-original reassortment viruses, rF/HA218G^+^/313G^+^ and rF/HA218G^−^/313G^+^, were generated. A functional glycosylation site in HA protein could affect on the mobility of HA protein on the gel [21]. The mobility analysis of HA proteins showed that the two potential glycosylation sites at amino acid residues 313 and 218 in HA protein are both functional glycosylation sites. The F/98 virus possesses a functional glycosylation site at amino acid residue 218. The rF/HA218G^+^/313G^+^ virus possesses two functional glycosylation sites at amino acid residues 218 and 313. The rF/HA218G^−^/313G^+^ virus possesses a functional glycosylation site at amino acid residue 313. And our result showed that the rF/HA218G^−^/313G^+^ viruses could bind to the antibody of the parental virus F/98 more efficiently than the viruses F/98, indicating that the rF/HA218G^+^/313G^+^ virus or the rF/HA218G^−^/313G^+^ virus could enhance antibody responses by increasing virus-antibody binding, or may compensate for the other mutants occurred in HA to maintain fitness during the evolution of H9N2 virus. NLG compensates for antibody escape fitness costs of antigenic escape mutations via a detrimental loss of binding avidity, which prevent binding of virus to host cells [15]. The antigenic profile of each virus is determined through HI assay using reference sera, and two factors could affect the HI titer, the receptor binding avidity, and antibody binding [22]. In this study, the receptor binding was free from variations in glycosylation sites at amino acid residues 313 and 218 (no date was shown). Additionally, our research team and other researchers find that the antibody selective pressure derived from vaccine affect the evolution of influenza virus, including variation of glycosylation sites [15, 33], and the time of changes on glycosylation sites at hemagglutinin amino acid 218 and 313 was similar to the time of changes on some antigenically sites on HA [34]. We hypothesize that the variations in the glycosylation sites at amino acid residues 313 and 218, which enhanced antibody responses by increasing virus-antibody binding, maybe reveal novel viral adaptive mechanisms. Furthermore, our results seem to demonstrate that changes on variations in glycosylation sites at amino acid residues 218 and 313 are associated with the viral antigenicity evolution. Additionally, growth of viruses lacking glycosylation at either 158N or 169N of the H5N1 avian influenza virus was significantly reduced both in MDCK and A549 cells, while replication of viruses with additional glycosylation 144N of H5N1 virus was significantly promoted, and mutant viruses with loss of 158N or 169N glycosylation sites showed increased pathogenicity in mice [24]. However, in our results, either the rF/HA218G^+^/313G^+^ virus or the rF/HA218G^−^/313G^+^ virus significantly increased infectivity of MDCK cells, chicken embryo eggs, and trachea and lung tissue in SPF chickens compared with F/98 virus, but they did not change the characteristics of the airborne spread in chickens or of infectivity of mice.

In conclusion, by analyzing and comparing the changes of glycosylation sites of the HA protein sequences in the H9N2 avian influenza viruses before 2017, we designed and generated two viruses, rF/HA218G^+^/313G^+^ virus and rF/HA218G^−^/313G^+^, based on the F/98 virus backbone, a 218G^+^/313G^−^ H9N2 avian influenza virus. In comparison with original F/98 virus, the variations in the glycosylation sites at amino acid residues 313 and 218 near the HA cleavage site increased antibody binding and significantly affected the viral infectivity of cells, chicken embryo eggs, or SPF chickens, but those changes did not affect the property of the airborne propagation, or infectivity of mice.
